# Influence of core color on final shade reproduction of zirconia crown in single central incisor situation – An *in vivo* study

**DOI:** 10.4317/jced.56401

**Published:** 2020-01-01

**Authors:** Ghada M. Ayash, Essam Ossman, Lucette G. Segaan, Mohammad Rayyan, Christelle Joukhadar

**Affiliations:** 1 Department of Oral Rehabilitation Sciences, Faculty of Dentistry, Beirut Arab University; 2 Clinical Instructor Department of Oral Rehabilitation Sciences, Faculty of Dentistry, Beirut Arab University

## Abstract

**Background:**

A lot of shaded zirconia blocks are being introduced into the market. Their effect on the final shade of the restoration is yet uncertain.

**Material and Methods:**

Twenty-four zirconia crowns were fabricated for 8 patients who needed to restore a single maxillary central incisor, and divided into 3 groups according to the color and type of the zirconia (Zr) used (white Zr core, colored Zr core, and monolithic high translucency (ht) Zr crowns). Using Easyshade spectrophotometer, delta E color difference was calculated between fabricated crown and adjacent tooth. The ΔEs obtained were assessed based on 1.6 ΔE which represented the color difference that could not be detected by the human eye and considered clinically acceptable.

**Results:**

No statistically significant values were found between the 3 groups related to different Zr color and type.

**Conclusions:**

Within the limitations of this study, it could be concluded that the shade of the zirconia blank had no significant effect on the final color of the crown. This raises reasonable doubt about the necessity to use colored zirconia blanks or use of dip-in solutions. The clinical implications were that, there was no need to use colored zirconia cores to get more esthetically pleasing restorations with respect to color perception. The use of monolithic high translucent zirconia crowns gained the advantages of high translucency and color reproduction.

** Key words:**Zirconia, easyshade, monolithic, spectrophotometer, delta E.

## Introduction

The replication of natural teeth, especially with single-tooth restorations, represents a challenge. Due to the fact of the individuality of each single human tooth, it is a great order to create single anterior restorations that match harmoniously to the existing adjacent teeth (1). Demands for highly esthetic restorations with advances in prosthetic fabrication processes and techniques have led to the introduction of zirconia crowns (2). The achievement of an all-ceramic esthetic restoration that matches perfectly with adjacent teeth is the result of the interplay between 2 important optical factors: on one hand, the masking capacity of ceramics to block the background color (in many cases, non-vital dentin or a core buildup material) with sufficient material thickness or an opaque masking liner, and on the other hand, the amount of translucency of the ceramic that will allow the natural background color shine throughout a translucent material and exhibit the most natural appearance. Different zirconia substructures have been suggested to improve the esthetic results of zirconia ceramic crowns (3). However, the final color result is unpredicTable when the restoration is composed of different layers with unspecified thicknesses, which is the case for core veneered all-ceramic restorations (4).

Contemporary high-strength zirconia-based ceramics exhibit very good clinical data and superior esthetics (5). Zirconia-crowns combine the benefits of metal restorations, such as minimally invasive tooth preparation and simple cementation, with those of all-ceramic crowns, such as low thermal conductivity and adequate translucency (6). However, one of the most challenging aspects of esthetic dentistry is color assessment and its reproduction (7). This specifically refers to the ability of dental ceramics to accurately match the color of the selected shade tab without the aid of intrinsic as well as extrinsic colorants (8). In ceramic crowns: there are 3 factors that can alter the final shade: 1-shade of the prepared tooth; 2- shade of crown itself; and 3- shade of the luting cement. These 3 components have to be optically harmonized to reproduce natural crown color. Assuming excellent color matching is verified in dental laboratory, it should be correctly transferred to the clinical setting (9). Zirconia frameworks are naturally white in color that may negatively influence the final esthetic appearance of the veneered restoration. This restricted the use of such restorations to posterior non-esthetic areas (10). In literature, it was presented that ceramics with high strength is likely to be more opaque and accordingly indicate more trials when demanding to match natural tooth color, but they can be used to mask discolorations when existing (11,12). Colored zirconia frameworks were introduced to the dental market to enhance color reproduction of ceramic restorations especially in cases with inadequate space to accommodate the required thickness of both framework and ceramic veneer.

Additionally, they could eliminate the use of masking liner material over white zirconia frameworks. Nevertheless, these new colored frameworks need adjustment of the veneer-application procedure to achieve the required final color. The bond strength between zirconia framework and ceramic veneer is affected by the coloring of the zirconia (13). To prevent delamination and chipping failures of zirconia veneered restorations, careful selection of both framework and veneer ceramics are essential to prevent chipping and delamination of bilayered zirconia restorations (14,15). Three major elements of color: hue, chroma and value are necessary to achieve accurate shade matching. However, a fourth element, translucency has become an important factor in precision of shade matching (16). Instrumental measurements of translucent restorative materials using spectrophotometer, colorimeter, spectroradiometer and digital camera plus software have been reported. Porcelain translucency is usually measured with the translucency parameter (TP) or the contrast ratio (CR) (17). Recently high translucency zirconia was introduced, with its promising performance to match lithium disilicate in esthetics and surpass it in strength. This material can be milled into full contour crowns. Prevention of delamination of the veneering ceramic using full contour translucent crowns in addition to preventing the high cost of the colored cores and its lengthy manipulation steps to achieve accurate bonding strength between the colored cores and the veneering ceramic become a question worth answering. Are colored zirconia blanks needed to produce esthetic crowns or does it really rely on the skill of the laboratory technician. The hypothesis of this study was: There will be a difference in color replication potential of different zirconia frameworks investigated.

## Material and Methods

Eight patients were recruited with the need to restore a single ceramic crown on one of their maxillary incisors. The patients were fully informed about the purposes and design of the clinical study and consent was obtained prior to treatment. The study design was accepted by the IRB committee at BAU (2014H-003-D-P-0012).

Patients were selected based on the following inclusion criteria:

• Indicated for single all-ceramic crowns on one of their maxillary central incisors.

• At least one of the adjacent teeth was sound to serve as a guideline and control for shade determination.

• Small proximal fillings that would not affect the buccal aesthetic appearance were accepted.

• Good oral hygiene and awareness.

For each patient 3 crowns were fabricated. Crowns were divided to 3 groups according to color and type of zirconia ( Table 1).

**Table 1:**
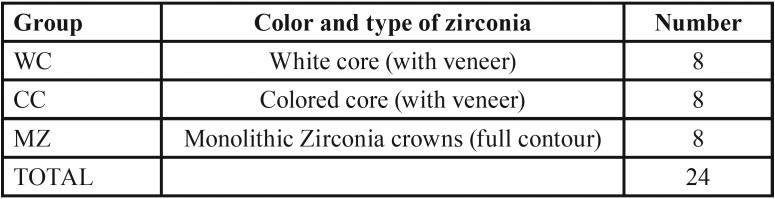
Sample Grouping.

All teeth were cleansed and polished with plain white dentifrice, using rubber-polishing cup on a low speed. Intraoral spectrophotometer (Easyshade V, Vita,Yorba Linda, North America) was used to record preoperative shades instrumentally for the central incisor to be restored and the target adjacent tooth as control shade. 

Each tooth underwent spectrophotometric analysis at circular areas of 1.5 mm on the middle of its labial surface with the probe tip positioned at 90 degrees to the surface of the tooth (18). Care was taken so that the probe was not moved during a measurement and/or set at a different angle. The specific area of the tooth was determined using a caliper to establish the midposition of the middle of the labial surface (19). Color quantification of the standardized circular area was based on CIE-Lab system and was expressed according to L*, a* and b* color parameters (20). Data was recorded 3 consecutive times. Following another calibration cycle, the entire process was repeated for the adjacent tooth. Three silicon indexes were made for each patient; one to aid in controlling the amount of tooth reduction incisally and axially, the second to help in fabricating a suiTable interim restoration and the third was sent to the laboratory for duplicating the full crown contour. A 0.9 mm sub gingival heavy chamfer finish line was prepared with 1.2 mm axial reduction, and 1.8 mm incisal clearance using round end tapered guided-pin diamond burs (21). Finishing of the preparations were performed to ensure smooth surfaces by the use of guided-pin finishing burs. Interim composite resin material was placed in the preformed index, placed over the prepared tooth until setting, removed, adjusted, and cemented with an eugenol-free temporary dental cement .

Final impressions were made in another session to give proper time for marginal periodontium healing and give time for interim restoration to mold the gingiva to prevent bleeding during retraction cord placement (22) using double cord for gingival displacement. After pouring in type IV stone and preparing accurate dies, they were scanned using Cercon eye scanner. Every patient received 3 zirconia crowns. Zirconia copings and monolithic crowns were produced by milling zirconia blanks and were sintered according to manufacturer instructions. For Group WC, 0.5 mm white zirconia core was milled from Cercon base light blank. For Group CC, 0.5mm colored zirconia core was milled from the Cercon base colored blanks. For Group MZ, full anatomical monolithic high translucent zirconia crowns were milled from Cercon ht blank by the use of Type 0 Cercon brain expert cutters and sintered at 1.500°C in the heat furnace.

For the 3 Groups, manual finishing after sintering was done to both the inner and outer surfaces of the copings using air particle abrasion with 100μ alumina particles. Each coping and crown were trial fitted to its corresponding tooth. Cleaning was accomplished by aid of a steam cleaner. Easyshade V was used for recording the color coordinates (L, a, and b) of each coping/crown. Copings were veneered using feldspathic ceramic (Cercon CeramS) using silicone index for group WC and CC whereas for group MZ, body stains were used as external stains on crowns.

Completed crowns were consecutively tried on corresponding patient for proper fit, contacts and occlusal adjustments (if needed) before they were sent for glazing.

Each patient was given the opportunity to select one of the 3 crowns to be cemented permanently on the prepared tooth. All patients selected the monolithic ones most probably for their light weight. All intaglio surfaces were air particle abraded using 100μ Al2O3 then zirconia primer was applied. Proper cementation procedures were accomplished by the use of the resin cement ( Multilink Automix, Ivoclar Vivadent AG, Schaan, Liechtenstein ).

Color Measurement

CIE color coordinates L*, a*and b* were calculated in each of 3 areas of the ceramic crowns. The areas of interest were measured 3x3 mm in the cervical, body, and incisal regions. Triplet measurements were performed, and average readings were used for data calculation. Color data obtained from the adjacent target tooth were considered as control. Color difference ΔE between the control (adjacent tooth) and the color of zirconia crowns was calculated as follows:

• ΔL*= L*control – L*experimental

• Δa*= a*control – a*experimental

• Δb*= b*control – b*experimental

• ΔE*= (ΔL*2+Δa*2+Δb*2)1/2

• The ΔEs obtained were assessed based on ΔE of 1.6 which represented the color difference that could not be detected by the human eye and considered clinically acceptable (9).

-Statistical Analysis

Results were recorded, tabulated, and statistically analyzed. Numerical data were explored for normality by checking the distribution of data, calculating the mean and median values as well as using tests of normality (Kolmogorov-Smirnov and Shapiro-Wilk tests). Color parameters (L*) and (b*) data showed parametric distribution while (a*) parameter, color change (ΔE) data showed non-parametric distribution. Data were presented as mean, standard deviation (SD), minimum, maximum and 95% confidence interval (95% CI) values.

For parametric data (L* and b*); repeated measures ANOVA test was used for comparisons between different groups and control group, to study the effect of core type on color parameters and to study the effect of cement on color parameters. Bonferroni’s post-hoc test was used for pair-wise comparisons when ANOVA test is significant.

For non-parametric data (a* and ΔE); Friedman’s test was used for comparisons between different groups and control group, to study the effect of core type and to study the effect of cement shade. Wilcoxon signed-rank test was used for pair-wise comparisons when Friedman’s test was significant. Bonferroni’s correction was applied for the pair-wise comparisons.

## Results

-Effect of core type on color parameters

In ( Table 2), as regards (L*) parameter using core alone; monolithic crown showed the statistically significant highest mean (L*) value (81.2 ± 3.9). White core showed statistically significant lower mean value (67.7 ± 6.2). Colored core, showed the statistically significant lowest mean (L*) value (55.9 ± 6.5). As regards (a*) parameter using core alone; colored core showed the statistically significantly highest mean (a*) value (7.3 ± 3.0). White cores, showed statistically significant lower mean value (4.0±3.1). Monolithic crowns, showed the statistically significant lowest mean (a*) (2.8 ± 1.3) ( Table 2). While for (b*) parameter using core alone; colored core showed the statistically significantly highest mean (b*) value (36.6 ± 5.2). Monolithic crown, showed statistically significantly lower mean value (31.0 ± 6.1). White core, showed the statistically significantly lowest mean (b*) value (18.8 ± 3.4) ( Table 2). While using core + veneer, core + veneer + A3 cement as well as core + veneer + Transparent cement; there was no statistically significant difference between the 3 core types for all the 3 parameters L*, a* and b*.

**Table 2:**
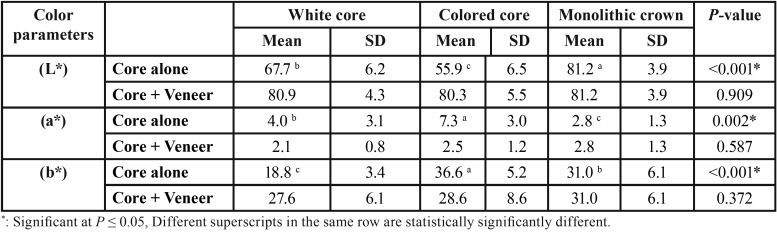
Comparison between color parameters of different core types.

-Color difference (ΔE)

 Descriptive statistics

**Figure 1:**
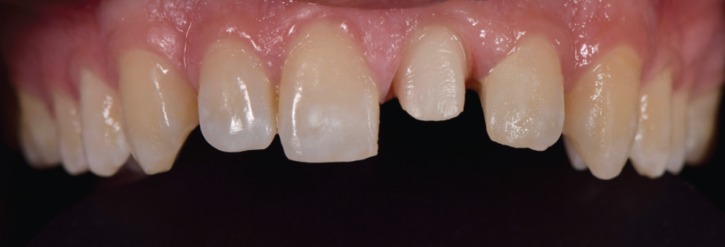
Tooth preparation.

**Figure 2:**
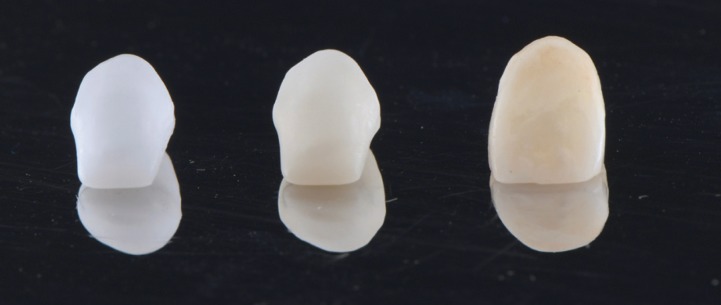
White Zr core, colored Zr core and ht Zr crowns.

**Figure 3:**
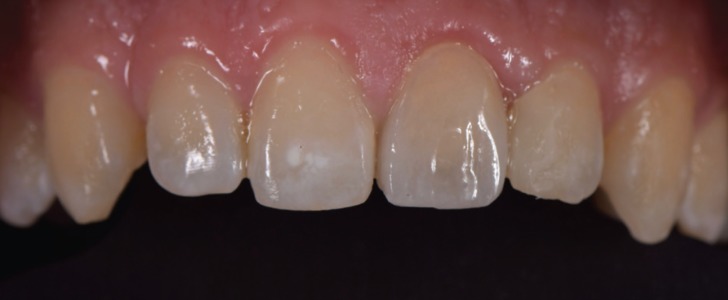
Finished ht Zr crown.

Descriptive statistics of color difference (ΔE) are presented in ( Table 3, Figs. 1-3).

**Table 3:**
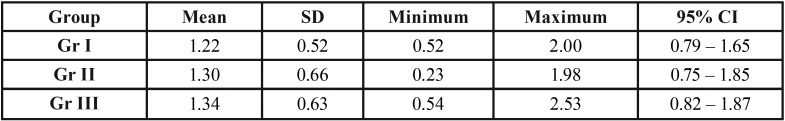
Comparison between color parameters of different core types.

## Discussion

In the present study, maxillary central incisor was chosen since as regards to esthetics, it poses the greatest restorative challenge for clinicians; not surprisingly, it can also be the most difficult tooth for the dental technician to match (23). Zirconia was the material of choice for its improved mechanical strength and unique stress-induced transformation toughening mechanism and proven biocompatibility (24,25).

Spectrophotometric color measurements in terms of the CIE L*, a*, and b* color coordinates for different Zr cores were reported. The color difference value (ΔE) represented the numerical distance between 2 colors each having the coordinates L*, a*, and b* (22). The purpose of the study, was to test the ability of different zirconia-based crowns milled from different blank shades to match the shade of the adjacent tooth and to calculate the color difference between the restoration and the natural control tooth.

“L” coordinate which represents the lightness of an object was (67.7) with the white core, (55.9) with the colored core, and (81.2) with the monolithic crown. The monolithic crown had the privilege being monolayer and the whole crown was milled from the same high translucency zirconia blank, this gave the bulk color more lightness. The white core was milled from the other blank, which was lighter than the colored blanks. In order to get the selected shade, darker colored blanks (than the selected shade) were chosen on purpose by the laboratory technician to achieve the desired shade by the addition of the lighter veneering ceramic. This justified the big difference between the “L” coordinate of the colored core alone (55.9) and that of colored core with veneering ceramic (80.3).

Veneering ceramic increased the “L” color coordinate of both the white and colored cores (80.9 and 80.3) respectively. For both white core alone or with veneer, the “L” color coordinates were always lighter than that of the colored cores or veneered colored cores. This finding came in accordance with Aboushelib *et al*, in 2010, who studied the influence of colored zirconia frameworks on the overall color match of zirconia-veneered restorations and found that white frameworks were too bright and offered high L values (10). Opposite to our study, Ozturk *et al.* in 2008 concluded that, as the ceramic thickness increased, significant reductions in L* values were recorded for IPS e-max Press and DC Zirkon specimens (27).

The authors compared the effects of various dentin ceramic thicknesses and repeated firings which may affected their results. In the current study the ceramic thickness was the same in all groups which secured the differences in color coordinates to be attributed to the difference in color as it was the only variable; also the number of firings wasn’t a tested variable.

The “a” color coordinate represents chromacity in which negative values indicate green and positive values indicate red. The highest mean of the “a” coordinate was found in the colored core (7.3) followed by the white core (4) and then the monolithic crown (2.8). The “b” color coordinate represents chromacity, with negative coordinates indicating blue and positive coordinates indicating yellow. As expected, the highest mean of the “b” coordinate was with the colored cores (36.6) compared to white core b mean 18.8. Monolithic crowns mean for “b” coordinate was 31. The values of the 3 color parameters (L*, a* and b*) were not statistically significant in between the tested groups. It was understood that, changing the zirconia color from white to colored or to monolith, didn’t give a significant change. ΔE values were statistically insignificant between the different zirconia crowns and the control group.

 It should be noted that although the ΔE values in current study fell within the perceivable range reported by Seghi *et al.* in 1989 (1 to 2 ΔE), it may not have a clinical relevance as far as color detection is concerned (28). That is because the ΔE values were lower than the accepTable range reported by Ishikawa-Nagai *et al.* in 2009 (1.6 ΔE) (9). This diversity in ΔE values between the various combinations, together with no detection of statistical significance in color parameter comparison, implies that the hard work that was usually paid upon the addition of metallic oxides and dip-in solutions to replace the dull white core is a bit questionable.

In our study, the CAD/CAM system used was the Cercon in which pre-colored zirconia blanks were available. No liner was used with the white zirconia cores before the addition of the veneering ceramic as recommended by the manufacturer’s instructions. This also helped in neglecting the effect of adding a liner on the final color of the white zirconia cores after veneering. Aboushelib *et al.* in 2010 reported that, the application of the required veneer ceramic over the natural zirconia framework resulted in an accurate reproduction of the required color; while for colored zirconia application of liner material or deep chroma dentin 

was necessary to reproduce the required color (10).

Their results were in accordance with current study, as the authors concluded that using pre-colored zirconia frameworks did not offer any direct advantages over the standard natural zirconia. Moreover, the selection of monolithic crowns and being more conservative in tooth preparation would rise to top by the insignificant color difference between the monolith and the other crowns.

Among the limitations of this study was the number of patients due to difficulty of finding patients in need to crown single central incisor.

A point worth mentioning was that most of the papers related to color coordinates, the specimens used were discs (29,30) whereas in current study crowns were used simulating the appropriate clinical conditions. Surprisingly, the color had no or minimal effect on the final shade of the crowns. It implies the necessity to purchase expensive colored blanks is no longer needed. The drawback of colored blanks on bond between zirconia core and veneer could be avoided.

The hypothesis was rejected as there was no difference in color among tested groups.
